# Elucidating the Role of Surface Ce^4+^ and Oxygen Vacancies of CeO_2_ in the Direct Synthesis of Dimethyl Carbonate from CO_2_ and Methanol

**DOI:** 10.3390/molecules28093785

**Published:** 2023-04-28

**Authors:** Guoqiang Zhang, Yuan Zhou, Yanlin Yang, Tiantian Kong, Ya Song, Song Zhang, Huayan Zheng

**Affiliations:** 1 Department of Food Science and Engineering, Moutai Institute, Renhuai 564502, China; 2 Experimental Training Teaching Center, Moutai Institute, Renhuai 564502, China

**Keywords:** carbon dioxide, dimethyl carbonate, CeO_2_, oxygen vacancy, lattice oxygen

## Abstract

Cerium dioxide (CeO_2_) was pretreated with reduction and reoxidation under different conditions in order to elucidate the role of surface Ce^4+^ and oxygen vacancies in the catalytic activity for direct synthesis of dimethyl carbonate (DMC) from CO_2_ and methanol. The corresponding catalysts were comprehensively characterized using N_2_ physisorption, XRD, TEM, XPS, TPD, and CO_2_-FTIR. The results indicated that reduction treatment promotes the conversion of Ce^4+^ to Ce^3+^ and improves the concentration of surface oxygen vacancies, while reoxidation treatment facilitates the conversion of Ce^3+^ to Ce^4+^ and decreases the concentration of surface oxygen vacancies. The catalytic activity was linear with the number of moderate acidic/basic sites. The surface Ce^4+^ rather than oxygen vacancies, as Lewis acid sites, promoted the adsorption of CO_2_ and the formation of active bidentate carbonates. The number of moderate basic sites and the catalytic activity were positively correlated with the surface concentration of Ce^4+^ but negatively correlated with the surface concentration of oxygen vacancies. The surface Ce^4+^ and lattice oxygen were active Lewis acid and base sites respectively for CeO_2_ catalyst, while surface oxygen vacancy and lattice oxygen were active Lewis acid and base sites, respectively, for metal-doped CeO_2_ catalysts. This may result from the different natures of oxygen vacancies in CeO_2_ and metal-doped CeO_2_ catalysts.

## 1. Introduction

The emission of CO_2_ into the earth’s atmosphere is continuously increasing with the growing burning of fossil fuels and industrial activities. For example, about 33,890.80 million tons of CO_2_ was emitted into the atmosphere in 2018. The global CO_2_ concentration in the atmosphere reached 407.65 ppm in September 2019, which is about 1.2 times as high as that of the past 40 years [1]. According to the earth’s CO_2_, the concentration of CO_2_ further increased to 416.47 ppm on 30 May 2020 [2]. The dramatically increased CO_2_ emission results in severe climate change, which brings about a series of issues in terms of ecology, economy, and the environment. Taking these facts into consideration, it is necessary to develop effective approaches to mitigate CO_2_ emission.

As an important part of carbon dioxide capture, utilization, and storage (CCUS), the chemical transformation of CO_2_ to fuels and value-added chemicals can not only alleviate CO_2_ emission but also reduce the dependence on fossil resources, which is of great significance for the sustainable development of energy, the environment, and the economy. Dimethyl carbonate (DMC), as one of the downstream products of methanol, is considered a green chemical, which has been widely applied as an intermediate for organic synthesis, an electrolyte for lithium-ion batteries, and a raw material for the synthesis of polycarbonate [3]. Among various synthesis approaches, the direct synthesis of DMC from CO_2_ and methanol is deemed a green route due to the beneficial environment impact and high atomic use [4].

Although this reaction suffers from low DMC yield due to the thermodynamic limit, a significant endeavor has been made to develop effective catalysts, such as supported catalysts, alkali carbonate catalysts, ionic liquid catalysts, heteropoly acid catalysts, and transition metal oxide catalysts [4,5]. Transition metal oxides, especially ZrO_2_ and CeO_2_, have attracted tremendous attention in recent years due to their promising catalytic activity. Fujimoto et al. [6] first reported that ZrO_2_ can catalyze CO_2_ and methanol to DMC, and the catalytic activity was closely related to the surface basic and acidic sites.

Bell et al. [7] further studied the reaction mechanism over ZrO_2_ using in situ infrared spectra. The results revealed that surface coordinately unsaturated Zr^4+^ cations, such as Lewis acid sites, promote the dissociation of methanol to methoxide (–OCH_3_) and a proton, which can react with the surface hydroxyl group to produce water. Next, bidentate carbonates were formed by the interaction of C and O atoms in CO_2_ with surface Lewis acid–base pairs (Zr^4+^–O^2−^), which were subsequently inserted into –OCH_3_ and formed monodentate methyl carbonate (MMC) intermediate species. Finally, the MMC species reacted with methyl (–CH_3_) from the dissociation of methanol on Lewis base sites (O^2−^) to produce DMC [7]. In this regard, the improvement of surface acidic and basic sites is beneficial for the catalytic activity. For example, Xiao et al. [8] found that the doping of Fe into ZrO_2_ can significantly improve the surface acidic and basic sites, and the catalytic activity was positively correlated with surface acidic and basic sites.

Compared with ZrO_2_, CeO_2_ shows much higher catalytic activity and thus receives much more attention. For example, Marin et al. [9] studied the kinetic and reaction mechanism over a CeO_2_ catalyst, and the results showed that the reaction follows the Langmuir–Hinshelwood mechanism, in which the adsorption of CO_2_ is considered the rate-determining step. Wang et al. [10] reported that the catalytic activity of CeO_2_ is closely related to the corresponding morphology, and CeO_2_ nanorods showed much higher catalytic activity than that of the cube and octahedron due to the exposure of more acidic and basic sites. A further study of the morphology effect was performed with in situ infrared spectra. The results indicated that the (110) facet of CeO_2_ nanorods promotes the formation of bidentate carbonates that can react with –OCH_3_ to form MMC. However, other carbonates, such as monodentate carbonates, bicarbonates, and bridged carbonates, cannot accomplish that process, and therefore, the bidentate carbonates were identified as active species in terms of CO_2_ adsorption [11]. In addition, doping the metal cation of Ca^2+^ [12], Zn^2+^ [13,14], Mn^2+^ [15], Ti^4+^ [16], Fe^3+^ [17], Co^4+^ [18], and Zr^4+^ [19,20] into the lattice of CeO_2_ promotes the formation of oxygen vacancies (O_V_) and is also proven to be an efficient strategy to improve catalytic activity. The results of in situ infrared spectra over the Zr–CeO_2_ catalyst demonstrated that O_V_ promote the formation of bidentate carbonates via the interaction of C and O atoms in CO_2_ with surface Lewis acid–base pairs (O_V_–O^2−^), in which one of the O atoms in CO_2_ is inserted into O_V_ [19]. Therefore, both the amount of adsorbed CO_2_ and the catalytic activity increase with the increasing concentration of O_V_ [12,13,14,15,16,17,18,19,20].

It is obvious that two different Lewis acid–base pairs, Zr^4+^–O^2−^ and O_V_–O^2−^, are considered active sites in ZrO_2_ and metal-doped CeO_2_ catalysts, respectively. However, of Ce^4+^ and O_V_, which one is catalytically active in CeO_2_ catalysts? Is the formation of O_V_ in CeO_2_ catalysts beneficial to the catalytic activity as in metal-doped CeO_2_ catalysts? The answers to these two questions are of prominent significance to the design of an effective catalyst. Inspired by this, the surface concentrations of Ce^4+^ and O_V_ were regulated via reduction and reoxidation of CeO_2_ under different conditions in this work. Combining XRD, TEM, XPS, CO_2_-TPD, NH_3_-TPD, and CO_2_-FTIR results, the role of surface Ce^4+^ and O_V_ in the adsorption of CO_2_ and the catalytic activity for DMC synthesis from CO_2_ and methanol were deeply elucidated. The possible influence of two types of O_V_ on the adsorption and activation of CO_2_ was also proposed.

## 2. Results

### 2.1. Textural Properties of Catalysts

**Figure 1:**
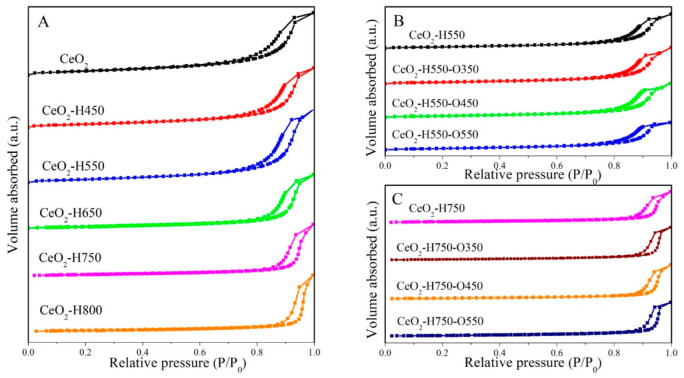
N_2_ adsorption–desorption isotherms of catalysts (**A**) CeO_2_ reduced at different temperatures, (**B**) CeO_2_–H550 reoxidized at different temperatures, and (**C**) CeO_2_–H750 reoxidized at different temperatures.

The N_2_ adsorption–desorption isotherms of CeO_2_ treated under different conditions are shown in Figure 1. All the samples showed the type IV isotherm with a distinct hysteresis loop, indicating the presence of a mesopore.

The detailed texture parameters are listed in Table 1. As seen in Table 1, the specific surface area and pore volume of CeO_2_ gradually decreased with an increase in the reduction temperature; however, the pore size of CeO_2_ presented the opposite trend, which suggests that the mesopore originated from the piled pore. This phenomenon may arise from the aggregation of CeO_2_ particles induced by high temperature [21]. In contrast, as CeO_2_–H550 and CeO_2_–H750 were reoxidized under an air atmosphere at 350 °C, 450 °C, and 550 °C, the texture parameters did not change so much.

### 2.2. XRD Analysis of Catalysts

**Figure 2:**
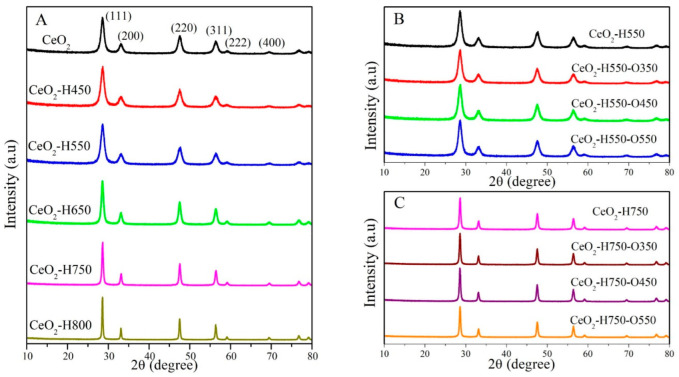
XRD patterns of catalysts (**A**) CeO_2_ reduced at different temperatures, (**B**) CeO_2_–H550 reoxidized at different temperatures, and (**C**) CeO_2_–H750 reoxidized at different temperatures.

XRD characterization was performed to analyze the crystal structure of CeO_2_, and the results are presented in Figure 2. All the samples displayed the characteristic diffraction peaks of a cubic fluorite structure (JCPDS no. 65-5923), and the peaks at 28.5°, 33.0°, 47.5°, 56.5°, 59.4°, and 69.6° were ascribed to the (111), (200), (220), (311), (222), and (400) crystal facets of CeO_2_, respectively [19].

As seen in Figure 2A, the intensity and width of the characteristic diffraction peaks of CeO_2_ did not change obviously as the reduction temperature decreased below 550 °C, indicating the similar crystal size of CeO_2_, CeO_2_–H450, and CeO_2_–H550. However, as the reduction temperature increased beyond 550 °C, the peak intensity was gradually enhanced and the peak width gradually narrowed, indicating the gradually increasing crystal size. The crystal size of each catalyst is presented in Table 2, which was calculated using the Scherrer equation as follows: *D = Kλ*/[(*β* − *b*)cos*θ*], where *K* is the Scherrer constant (0.89), *λ* is 0.15414 nm (the wavelength of Cu K*α* radiation), *β* is the full width at half-maximum of the CeO_2_(111) diffraction peak, *b* is the corrected full width at half-maximum of the instrument, and *θ* is the diffraction angle [22].

As seen in Table 2, similar crystal sizes of about 7~8 nm were observed for CeO_2_, CeO_2_–H450, and CeO_2_–H550. However, the crystal sizes significantly increased from 7.4 to 46.6 nm with an increase in the reduction temperature from 550 °C to 800 °C, which resulted from the aggregation of CeO_2_ particles [21,23,24]. In addition, the crystal sizes of CeO_2_–H550 remained basically about 7~8 nm after reoxidation treatment, while those of CeO_2_–H750 remained basically about 30 nm after reoxidation treatment. These results revealed that reoxidation treatment does not obviously affect the crystal sizes of CeO_2_.

### 2.3. TEM of Catalysts

**Figure 3:**
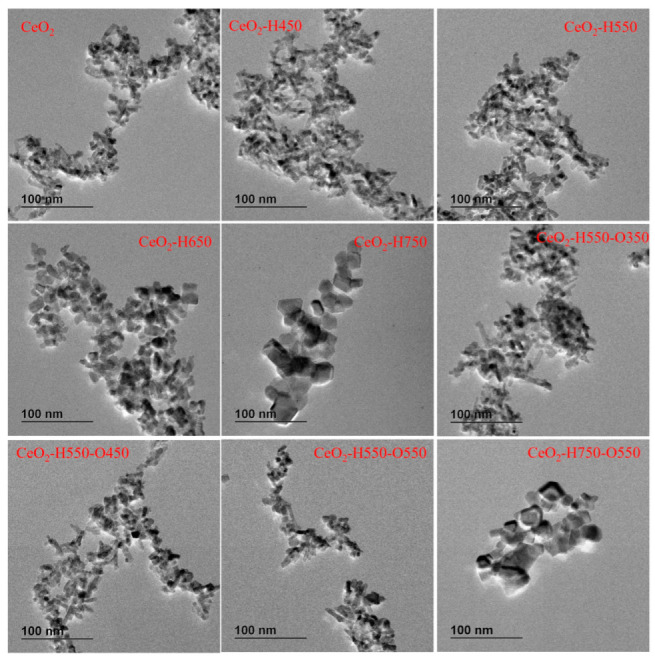
Representative TEM images of catalysts.

TEM was used to characterize the morphology of catalysts. As seen in Figure 3, the morphology of catalysts was irregular, including being polyhedron- and rodlike. The particle size of CeO_2_, CeO_2_–H450, CeO_2_–H550, CeO_2_–H550–O350, CeO_2_–H550–O450, and CeO_2_–H550–O550 seemed almost the same. The possible reason may be that the starting material of CeO_2_ was already calcinated at 550 °C for 5 h in a muffle furnace before reduction and reoxidation treatment. However, the particles significantly aggregated with increasing reduction temperature as the reduction temperature increased beyond 550 °C. In addition, reoxidation treatment did not obviously affect the particle sizes of the catalysts. This was consistent with the results of XRD. 

**Figure 4:**
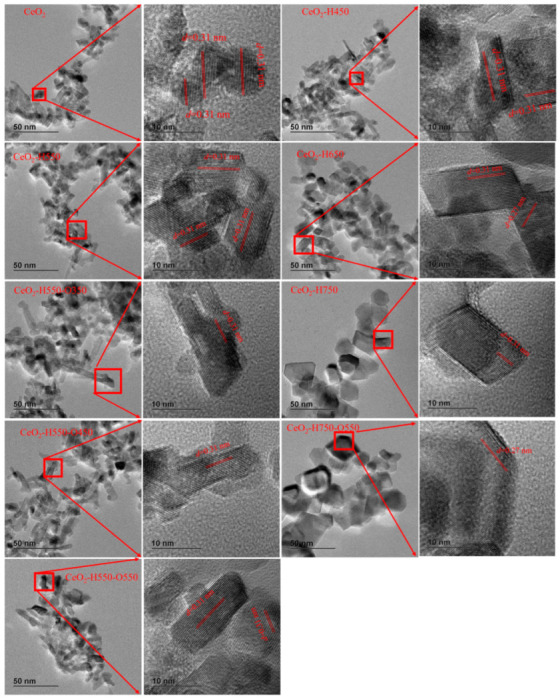
Representative HRTEM images of catalysts.

HRTEM was used to further characterize the exposed facet of catalysts, as shown in Figure 4. The observed lattice fringes of about 0.31 and 0.27 nm corresponded to the (111) and (200) facets of CeO_2_. Obviously, the main exposed facet was (111), and the (200) facet only appeared in CeO_2_–H650 and CeO_2_–H750–O550, which was in line with our previous studies [15,19]. These results also indicated that the reduction temperature and reoxidation treatment do not apparently affect the exposed facet of catalysts.

### 2.4. Surface Composition Analysis of Catalysts

**Figure 5:**
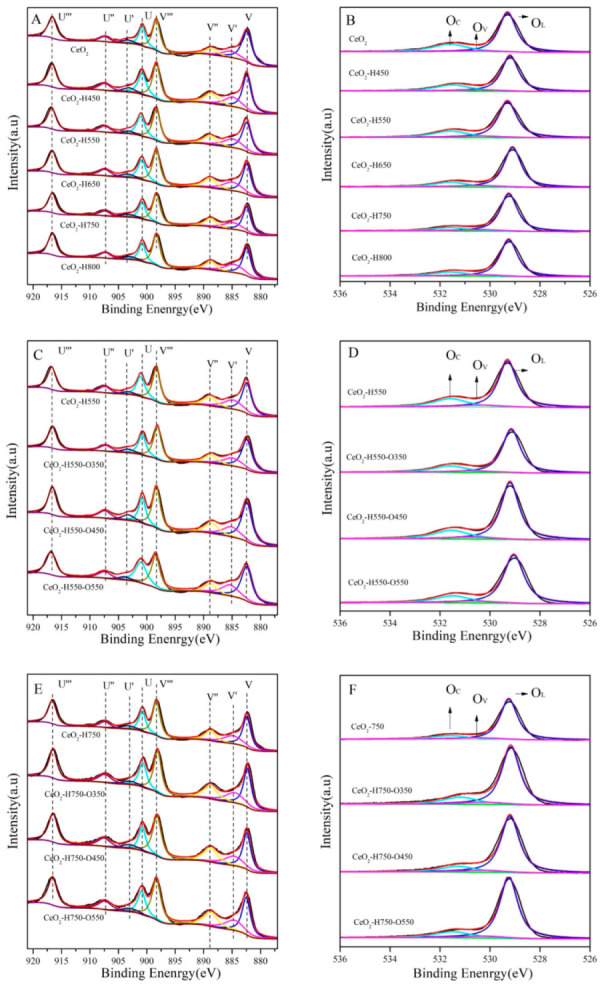
Ce 3d (**A**) and O 1s (**B**) XPS spectra of CeO_2_ reduced at different temperatures, Ce 3d (**C**) and O 1s (**D**) XPS spectra of CeO_2_–H550 reoxidized at different temperatures, and Ce 3d (**E**) and O 1s (**F**) XPS spectra of CeO_2_–H750 reoxidized at different temperatures.

XPS characterization was performed to analyze the chemical state of the surface Ce and O of catalysts, as shown in Figure 5. After gaussian fitting, the Ce 3D spectra could be fitted into eight peaks: U‴(~916.3 eV), U″(~907.4 eV), U′(~903.3 eV), U(~900.6 eV), V‴(~898.0 eV), V″(~888.4 eV), V′(~884.9 eV), and V(~882.1 eV) [16].

In the O 1s spectra, the peaks at ~529.0 eV, ~530.5 eV, and ~531.5 eV were assigned to the surface lattice oxygen (O_L_), oxygen vacancies (O_V_), and chemisorbed oxygen species (O_C_), respectively [25,26]. The surface concentration of O_V_ was calculated based on the peak area from gaussian fitting, and the results are listed in Table 3. 

As seen in Table 3, the concentration of Ce^3+^ gradually increased from 13.5 to 17.7% as the reduction temperature increased to 800 °C, while that of Ce^4+^ gradually decreased from 86.5 to 82.3%. Moreover, the concentration of O_V_ also increased with an increase in the reduction temperature. These results were consistent with the work reported by our group and Chen et al. that the percentage of Ce^3+^ is positively correlated with that of O_V_ [16,19]. In addition, the concentrations of Ce^3+^ and O_V_ decreased for CeO_2_–H550 and CeO_2_–H750 after reoxidation treatment.

The concentration of the surface O_V_ of CeO_2_ is closely related to the calcination atmosphere, oxygen partial pressure, and calcination temperature [27]. The formation of O_V_ is accomplished through the diffusion of lattice oxygen to the environment, where the content of oxygen is low. Under these circumstances, an oxygen vacancy is a kind of anionic defect. When CeO_2_ was exposed to the reductive H_2_ atmosphere, the lattice oxygen reacted with H_2_ and formed H_2_O and therefore promoted the formation of O_V_; moreover, the promoting effect gradually strengthened with increasing reduction temperature. It should be noted that two surplus electrons would be retained in the crystal structure of CeO_2_ accompanied with the formation of O_V_, and the two surplus electrons can bind with two adjacent Ce^4+^, resulting in the conversion of Ce^4+^ to Ce^3+^. Moreover, the two electrons were mobile that could migrate between two adjacent Ce ions. In other words, the two quasi-free mobile electrons were trapped around the anionic defect (O_V_), which could migrate between adjacent Ce ions and cause electron conduction. A material with this kind of defect is an N-type semiconductor [28]. The O atoms were reinserted back into O_V_ and formed O_L_ when CeO_2_–H550 and CeO_2_–H750 were reoxidized under an air atmosphere. In this process, the trapped electrons around O_V_ were transferred to O atoms, and the conversion of Ce^3+^ to Ce^4+^ occurred simultaneously. Singhal et al. [29] also found that the conversion between O_V_ and O_L_ is reversible to some extent.

Furthermore, as shown in Figure S1, the color of CeO_2_ gradually changed from yellow to brown and finally to gray-black with the increase in the reduction temperature, and the color changed back to yellow to a certain degree when CeO_2_–H550 and CeO_2_–H750 were reoxidized under an air atmosphere. This is because the trapped electrons around O_V_ can absorb light with a certain wavelength and the absorption capacity is gradually enhanced with the increasing number of electrons or O_V_ [21,29]. This result also indicated that reduction treatment is favorable for the formation of O_V_, while reoxidation treatment plays the opposite role.

### 2.5. Surface Acidity and Basicity Analysis of Catalysts

**Figure 6:**
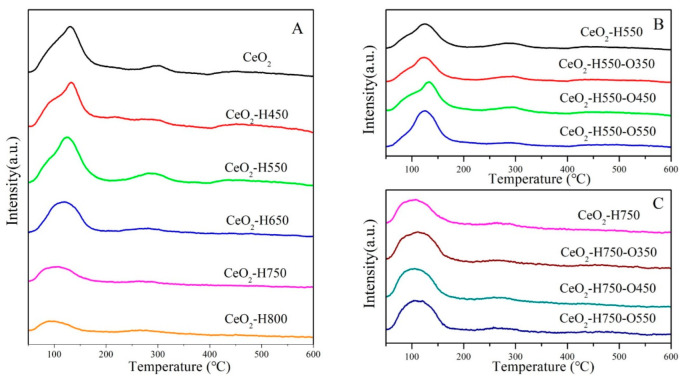
NH_3_-TPD profiles of catalysts (**A**) CeO_2_ reduced at different temperatures, (**B**) CeO_2_–H550 reoxidized at different temperatures, and (**C**) CeO_2_–H750 reoxidized at different temperatures.

**Figure 7:**
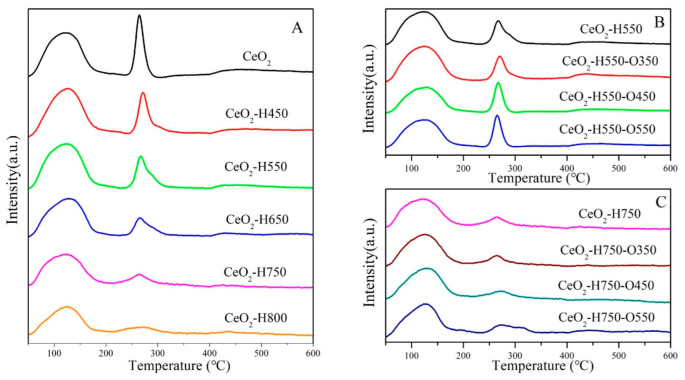
CO_2_TPD profiles of catalysts (**A**) CeO_2_ reduced at different temperatures, (**B**) CeO_2_–H550 reoxidized at different temperatures, and (**C**) CeO_2_–H750 reoxidized at different temperatures.

The surface acidity and basicity of catalysts were characterized using NH_3_-TPD and CO_2_-TPD, as shown in Figure 6 and Figure 7, and the detailed results are listed in Table 4. The desorption peaks in the temperature region of 50~200, 200~400, and 400~600 °C in CO_2_-TPD and NH_3_TPD profiles were attributed to the weak acidic/basic, moderate acidic/basic, and strong acidic/basic sites, respectively [30]. Studies have reported that weak basic sites are mainly ascribed to surface –OH groups, which react with CO_2_ and form bicarbonates [31,32]. The moderate basic sites were derived from the Ce–O_L_ or O_V_–O_L_ ion pairs, which combined with CO_2_ and promoted the formation of bridged and bidentate carbonates [8,19,32], while the strong basic sites were mainly assigned to the low-coordination oxygen anions [8,31,32], which resulted in the formation of stable monodentate carbonates [33].

Overall, the surface acidic sites with different natures all decreased with an increase in the reduction temperature and increased with an increase in the reoxidation temperature (Figure 6 and Table 4). A similar trend was observed for the surface basic sites under various treatment conditions (Figure 7 and Table 4). A possible reason for the change in surface acidity and basicity is discussed in Section 3.2.

### 2.6. Measurement of Catalytic Activity

**Figure 8:**
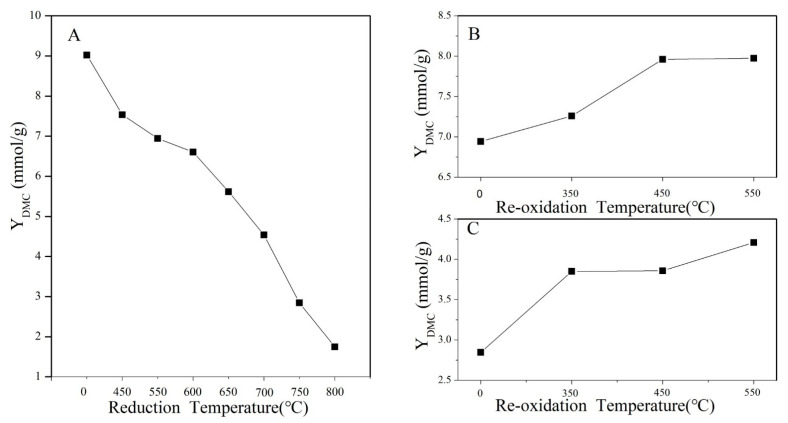
DMC yield of catalysts (**A**) CeO_2_ reduced at different temperatures, (**B**) CeO_2_–H550 reoxidized at different temperatures, and (**C**) CeO_2_–H750 reoxidized at different temperatures.

The DMC yields of CeO_2_ treated under different conditions are displayed in Figure 8. As seen in Figure 8A, the DMC yield of CeO_2_ gradually decreased from 9.02 to 1.75 mmol/g as the reduction temperature increased to 800 °C. However, as seen in Figure 8B,C, the DMC yield of CeO_2_–H550 and CeO_2_–H750 gradually increased from 6.94 and 2.85 to 7.98 and 4.21 mmol/g, respectively, as the reoxidation temperature increased to 550 °C. These results indicated that reduction treatment is detrimental to the improvement of catalytic activity but reoxidation treatment is beneficial.

## 3. Discussion

### 3.1. The Relationship between Catalytic Activity and Surface Acidity/Basicity

**Figure 9:**
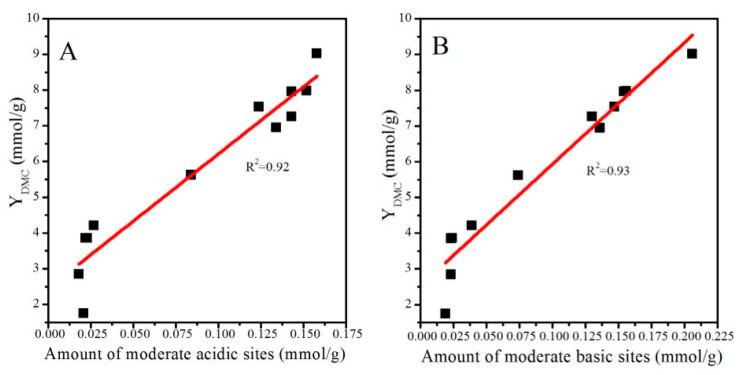
Relationships between catalytic activity and the number of moderate acidic (**A**) and basic (**B**) sites.

Surface basicity and acidity play a significant role in determining the catalytic activity of CeO_2_-based catalysts. Wang et al. [11] found that only bidentate carbonates can react with surface –OCH_3_ originating from the dissociation of methanol and form MMC, which is considered an active intermediate species for the formation of DMC, while monodentate carbonates, bicarbonates, and bridged carbonates cannot accomplish this process. Inumaru et al. [34] also found that the catalytic activity of ZrO_2_ nanocrystals is closely related to the surface bidentate carbonate species. As discussed in Section 2.4, bidentate carbonates were derived from the interaction of CO_2_ with moderate basic sites. In addition, surface acidic sites significantly affected the adsorption and activation of methanol. Studies have reported that methanol is adsorbed and dissociated on acid sites and forms –OCH_3_ groups, which participate in the formation of MMC [7,19,35]. Xiao et al. [8] revealed that catalytic activity is positively correlated with the moderate surface acidic/basic sites of Fe–Zr composite catalysts. Taking these findings into account, it is reasonable to build a relationship between catalytic activity and moderate surface acidic/basic sites. As shown in Figure 9A,B, catalytic activity was positively correlated with the number of moderate surface acid and basic sites (determined from NH_3_-TPD and CO_2_TPD profiles) of CeO_2_ catalysts. 

### 3.2. Deep Insight into the Role of Ce^4+^ and O_V_

Marin et al. [9] reported that conversion of CO_2_ and methanol to DMC over a CeO_2_ catalyst follows a Langmuir–Hinshelwood mechanism, with the CO_2_ adsorption step being rate controlling. However, there are two different theories about the adsorption and activation of CO_2_ for CeO_2_-based catalysts. One is that surface coordination unsaturated Ce^4+^ ions and O_L_ serve as Lewis acid–base pairs to promote the adsorption and activation of CO_2_ [7,36,37]. The other is that surface O_V_ and O_L_ serve as Lewis acid–base pairs to promote the adsorption and activation of CO_2_. Especially for metal-doped CeO_2_ catalysts, it was found that one of the O atoms of CO_2_ can be inserted into O_V_ and thus promotes the adsorption and activation of CO_2_, and therefore, the adsorption of CO_2_ improves with an increase in surface O_V_ [12,15,19]. Obviously, the focus was that of Ce^4+^ or O_V_, which Lewis acid sites determine the adsorption and activation of CO_2_ for CeO_2_ catalysts?

XPS results showed that reduction treatment results in a decrease in surface Ce^4+^ and an increase in O_V_. In contrast, reoxidation treatment resulted in a decrease in surface O_V_ and an increase in Ce^4+^. Combining XPS and CO_2_-TPD results, it was apparent that the adsorption amount of CO_2_ and the concentration of Ce^4+^ showed a similar trend under various treatment conditions. Moreover, combining XPS and NH_3_TPD results, we found that the adsorption amount of NH_3_ and the concentration of Ce^4+^ shows a similar trend under various treatment conditions. These results indicated that surface Ce^4+^ serves as active Lewis acid sites to adsorb and activate CO_2_ and methanol molecules.

Nevertheless, it should be noted that the particle size of CeO_2_ can also affect the adsorption of CO_2_ and NH_3_. For example, as the reduction temperature increased beyond 550 °C, the aggregation of CeO_2_ particles occurred (Table 2 and Figure 3), which can also result in decreased adsorption of CO_2_ and NH_3_. 

**Figure 10:**
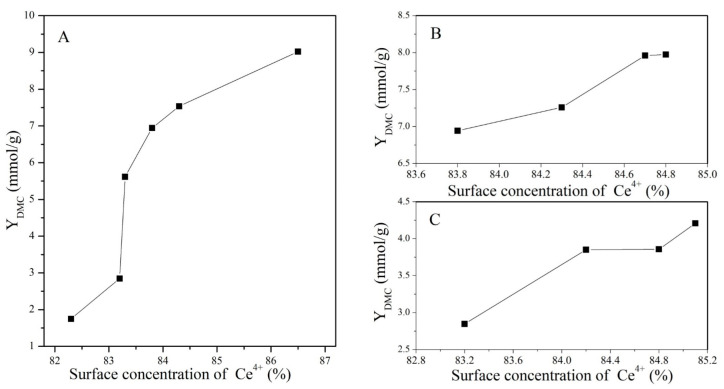
Variation in DMC yield with the surface concentration of Ce^4+^: (**A**) CeO_2_ reduced at different temperatures, (**B**) CeO_2_–H550 reoxidized at different temperatures, and (**C**) CeO_2_–H750 reoxidized at different temperatures.

**Figure 11:**
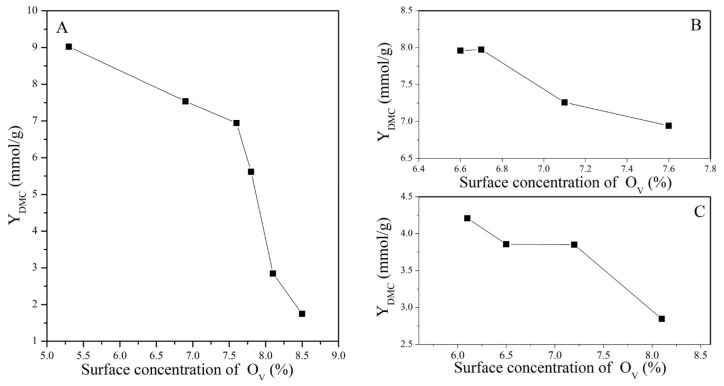
Variation in DMC yield with the surface concentration of O_V_: (**A**) CeO_2_ reduced at different temperatures, (**B**) CeO_2_–H550 reoxidized at different temperatures, and (**C**) CeO_2_–H750 reoxidized at different temperatures.

The profiles of the DMC yield for catalysts treated under different conditions with a change in the concentration of Ce^4+^ and O_V_ are presented in Figure 10 and Figure 11, respectively.

**Figure 12:**
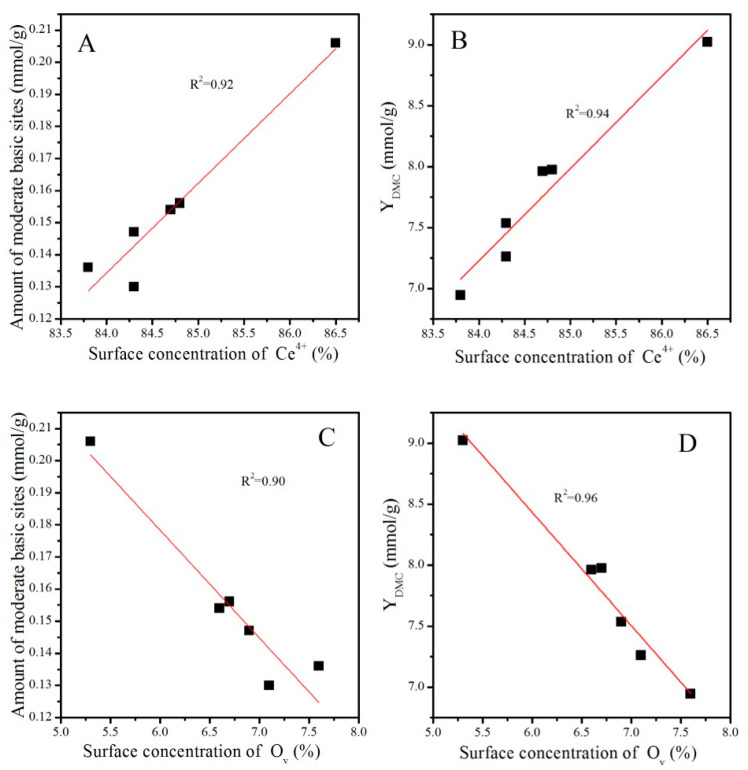
Correlations of the number of moderate basic sites and catalytic activity with the surface concentrations of Ce^4+^ (**A**,**B**) and O_V_ (**C**,**D**).

It was obvious that the catalytic activity increased with the increasing concentration of Ce^4+^, while it decreased with the increasing concentration of O_V_. Nevertheless, as discussed before, treatment conditions greatly affected the particle size of CeO_2_, as presented in Table 2 and Figure 3. Generally, a particle with a smaller size is conducive to the exposure of more active sites and thus improves the adsorption of the reactant and catalytic activity [38]. To eliminate the particle size effect, CeO_2_, CeO_2_–H450, CeO_2_–H550, CeO_2_–H550–O350, CeO_2_–H550–O450, and CeO_2_–H550–O550 catalysts (with similar crystal size of about 7~8 nm and basically an unchanged particle size) were selected to correlate the number of moderate basic sites as well as catalytic activity with the concentration of Ce^4+^ and O_V_. As shown in Figure 12, the number of moderate basic sites as well as catalytic activity were positively correlated with the concentration of surface Ce^4+^ but negatively correlated with that of O_V_. 

**Figure 13:**
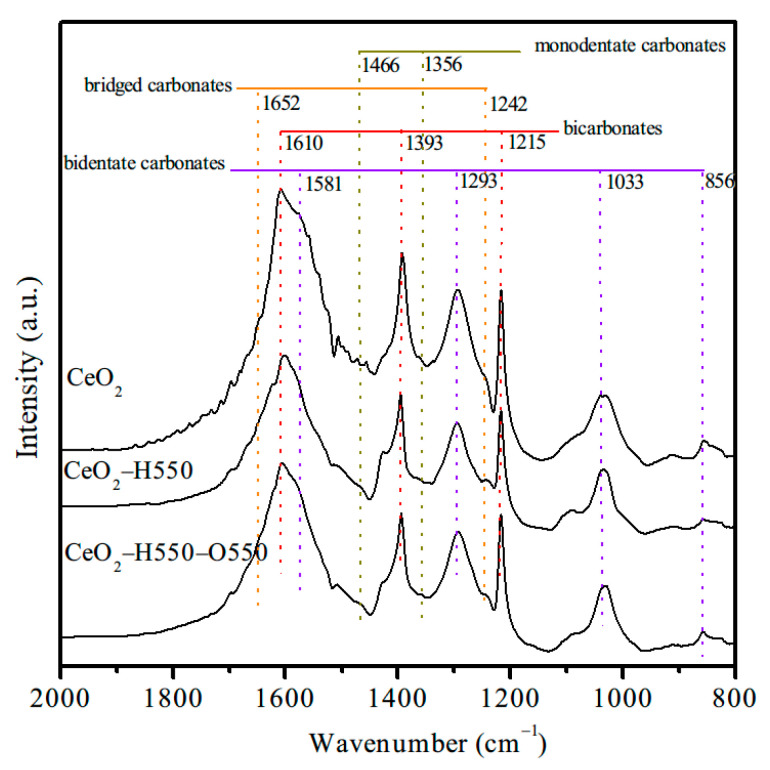
FTIR spectra of adsorbed CO_2_ on CeO_2_, CeO_2_–H550, and CeO_2_–H550–O550.

To further confirm the role of surface Ce^4+^ in the formation of active bidentate carbonates, FTIR spectra of CO_2_ adsorption for CeO_2_, CeO_2_–H550, and CeO_2_–H550–O550 were developed, and the results are displayed in Figure 13. The bands at 1215, 1393, and 1610 cm^−1^ were associated with bicarbonates, while the bands at 856, 1033, 1293, and 1581 cm^−1^ were ascribed to bidentate carbonates [11,19]. In addition, the small bands at 1242 and 1652 cm^−1^ were attributed to bridged carbonates, while the small bands at 1356 and 1466 cm^−1^ were assigned to monodentate carbonates [11,19]. Obviously, the peak intensity and area of bidentate carbonates decreased after reduction treatment and increased after reoxidation treatment. These results strongly supported the fact that the surface Ce^4+^ of CeO_2_ serves as active Lewis acid sites and promotes the adsorption of CO_2_ and the formation of bidentate carbonates and thus catalytic activity for the synthesis of DMC from CO_2_ and methanol.

**Figure 14:**
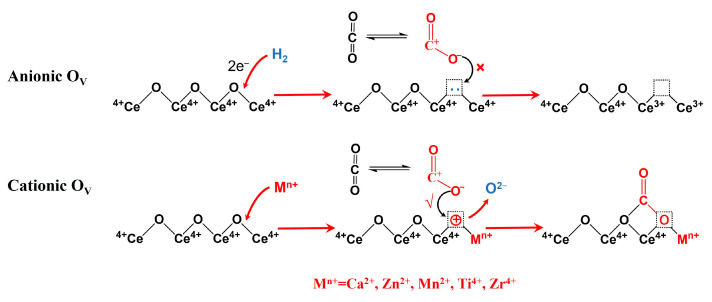
Possible influence of two types of O_V_ on the adsorption and activation of CO_2_.

Why O_V_ played an opposite role in the adsorption of CO_2_ for pure CeO_2_ and metal-doped CeO_2_ catalytic systems is thought provoking. This may be because O_V_ has a different nature in pure CeO_2_ and metal-doped CeO_2_ catalysts. As discussed in Section 2.3, the O_V_ formed by H_2_ reduction are a kind of anionic defect, and a material with this kind of defect is an N-type semiconductor [28]. The possible reason is that the quasi-free mobile electrons around O_V_ impede the adsorption and activation of CO_2_, and therefore, the catalytic activity decreases with an increase in O_V_. This was consistent with the results reported by Tan et al. that the catalytic activity of CeO_2_ prepared under different atmospheres follows the order CeO_2_–O_2_ > CeO_2_–Ar > CeO_2_–air > CeO_2_–H_2_; especially, the catalytic activity of CeO_2_–O_2_ was 2.5 times as high as that of CeO_2_–H_2_ [27]. However, for metal-doped CeO_2_ catalysts, the O_V_ derived from the incorporation of metal ions (for example, Ca^2+^, Zn^2+^, Mn^2+^, Ti^4+^, and Zr^4+^) into the lattice of CeO_2_ is a kind of cationic defect, and a material with this kind of defect is a P-type semiconductor[39,40]. This kind of cationic defect (O_V_) is beneficial for the adsorption and activation of CO_2_, and thus, the catalytic activity increases with an increase in O_V_ [12,13,15,16,19]. The possible influence of two types of O_V_ on the adsorption and activation of CO_2_ is shown in Figure 14. 

It is interesting that similar phenomena have also been observed during the conversion of syngas to light olefins over ZrCeZnO_x_ catalysts [41,42]. It was found that the incorporation of Ce^3+^ into Zn–Zr oxide improves the percentage of O_V_ and thus catalytic activity [42]; however, the catalytic activity of ZrCeZnO_x_ decreased after reduction with H_2_, although the percentage of O_V_ improved significantly [41]. This indicated that the types of O_V_ remarkably affects the adsorption of CO and therefore catalytic activity.

## 4. Materials and Methods

### 4.1. Preparation of CeO_2_

First, 3.47 g of Ce(NO_3_)_3_·6H_2_O was dissolved in 30 mL of deionized water. Next, 100 mL of NaOH solution (2 mol/L) was added to the solution of Ce(NO_3_)_3_ with a constant-flux pump under stirring. After aging for 1 h at ambient temperatures, the slurry was filtered and washed with deionized water and ethanol several times until it became neutral. Finally, the products were dried for 12 h at 80 °C and then calcinated for 5 h at 550 °C in a muffle furnace. The resultant catalyst was labeled as CeO_2_ and used as a starting material for further treatment.

### 4.2. Reduction Treatment of CeO_2_

Typically, 0.25 g of CeO_2_ was placed in a tube furnace, and then, the temperature was increased to 450, 550, 600, 650, 700, 750, and 800 °C under 10a % H_2_–N_2_ atmosphere for 4 h at a heating rate of 5 °C/min. The obtained catalysts were named as CeO_2_–HT_1_, where T_1_ is the reduction temperature.

### 4.3. Reoxidation Treatment of CeO_2_–H550 and CeO_2_–H750

Typically, 0.2 g of CeO_2_–H550 or CeO_2_–H750 was placed in a muffle furnace, and then, the temperature was increased to 350, 450 and 550 °C under an air atmosphere for 4 h at a heating rate of 2 °C/min. The obtained catalysts were named as CeO_2_–H550–OT_2_ or CeO_2_–H750–OT_2_, where T_2_ is the reoxidation temperature.

### 4.4. Characterization of Supports and Catalysts

N_2_ physisorption was performed on a Beishide 3H-2000PS2 instrument at −196 °C using N_2_ as an adsorbate. The sample was outgassed at 250 °C for 4 h in vacuum to remove the physisorbed impurities before measurement. The specific surface area and pore volume were calculated using the BET and BIH methods, respectively.

XRD characterization was carried out on a Rigaku SmartLab SE diffractometer (40 kV, 40 mA) with a diffraction angle (2*θ*) from 10 to 80° and a scanning rate of 10°/min. 

TEM images were taken with FEI Tecnai F30 at 200 kV. Before measurement, the sample was first dispersed in ethanol, then ultrasonicated for 30 min, and finally deposited on a carbon-coated copper film. 

TPD characterization was performed on Micromeritics Autochem 2920. First, 100 mg of the sample was degassed for 30 min at 250 °C with N_2_ (30 mL/min) to remove the physisorbed impurities. After the temperature was cooled down to 50 °C, 15% NH_3_ in He (30 mL/min) or pure CO_2_ (30 mL/min) was introduced and maintained for 30 min to allow saturated adsorption of NH_3_ and CO_2_. Subsequently, the physisorbed substance was removed with He (30 mL/min) at 50 °C for 30 min. Thereafter, the temperature was gradually increased to 800 °C at a heating rate of 10 °C/min with He as a carrier gas (30 mL/min), and the desorbed NH_3_ and CO_2_ were detected with TCD.

XPS spectra were analyzed with an ESCALAB 250Xi (Thermo Scientific, Waltham, MA, USA) photoelectron spectrometer under AlKα radiation at 300 W. The binding energies were referred to the C1s line from adventitious carbon at 284.6 eV.

CO_2_-FTIR spectra were recorded using a Bruker Tensor II instrument equipped with an MCT detector by collecting 64 scans at a resolution of 4 cm^−1^. About 20 mg of the sample was loaded into the cell and then pretreated under He flow (30 mL/min) at 400 °C for 1 h to remove the physisorbed impurities. After the temperature dropped down to 140 °C (reaction temperature), pure CO_2_ (30 mL/min) was introduced and maintained for 30 min to allow saturated adsorption of CO_2_. Subsequently, the physisorbed substance was removed with He (30 mL/min) for 30 min, and then, FTIR spectra were recorded.

### 4.5. Catalytic Evaluation

The catalytic activity of catalysts for DMC synthesis from CO_2_ and methanol was investigated in a 250 mL high-pressure slurry-bed reactor equipped with a mechanical stirrer. Typically, 0.2 g of a catalyst and 30 mL of methanol were placed into the reactor, which was subsequently sealed. Next, the reactor was filled with CO_2_ several times to check the tightness and remove air. Thereafter, the reactor was pressurized with CO_2_ to 3 MPa at ambient temperatures, and then, the reaction temperature was increased to 140 °C and maintained for 4 h. After the reaction finished, the reactor was depressurized and cooled down to room temperature. The liquid product was analyzed with n-propanol as an internal standard substance using a gas chromatograph (FID-GC, O Hua 9160). It should be noted that no by-products were formed except DMC, and the yield of DMC was defined as follows:

(1) $$Y_{\mathrm{DMC}}\left(\mathrm{mmol}/g\right)=n_{\mathrm{DMC}}(\mathrm{mmol})/m_{\mathrm{catalyst}}(g)$$

## 5. Conclusions

(1) The concentrations of surface Ce^4+^ and oxygen vacancies of CeO_2_ were regulated with reduction and reoxidation treatments under different conditions. The reduction treatment promoted the conversion of Ce^4+^ to Ce^3+^ and improved the surface concentration of oxygen vacancies, while the reoxidation treatment favored the conversion of Ce^3+^ to Ce^4+^ and decreased the concentration of oxygen vacancies.
(2) Catalytic activity was positively correlated with the number of moderate surface acidic/basic sites of catalysts. Moreover, the number of moderate basic sites and catalytic activity were positively correlated with the surface concentration of Ce^4+^ but negatively correlated with that of oxygen vacancies.
(3) The surface Ce^4+^ rather than oxygen vacancies served as active Lewis acid sites, and lattice oxygen served as active Lewis base sites to adsorb and activate CO_2_, promoting the formation of active bidentate carbonates species and DMC.
(4) The cationic oxygen vacancy was beneficial but the anionic oxygen vacancy was detrimental to the formation of active bidentate carbonates species and DMC, which sheds light upon the active sites for CeO_2_ and metal-doped CeO_2_ catalysts and provides evidence for the design of efficient catalysts.

## Figures and Tables

**Table 1:** Textural parameters of catalysts.

Catalysts	S_BET_ (m^2^·g^−1^)	V_p_ (cm^3^/g)	Pore Size (nm)
CeO_2_	130.3	0.65	13.8
CeO_2_–H450	120.9	0.59	14.6
CeO_2_–H550	106.2	0.57	14.6
CeO_2_–H650	72.9	0.47	16.9
CeO_2_–H750	28.2	0.27	23.1
CeO_2_–H800	18.6	0.24	30.7
CeO_2_–H550–O350	107.3	0.59	14.8
CeO_2_–H550–O450	109.2	0.64	14.9
CeO_2_–H550–O550	107.8	0.52	12.3
CeO_2_–H750–O350	23.1	0.23	25.1
CeO_2_–H750–O450	26.2	0.27	25.6
CeO_2_–H750–O550	24.5	0.24	23.9

**Table 2:** Crystal and particle size of catalysts.

Catalysts	*d*_CeO_2__ (nm) ^a^	Catalysts	*d*_CeO_2__ (nm) ^a^
CeO_2_	7.1	CeO_2_–H550–O350	7.6
CeO_2_–H450	7.4	CeO_2_–H550–O450	7.3
CeO_2_–H550	7.4	CeO_2_–H550–O550	7.5
CeO_2_–H650	12.9	CeO_2_–H750–O350	32.1
CeO_2_–H750	25.5	CeO_2_–H750–O450	29.7
CeO_2_–H800	46.6	CeO_2_–H750–O550	31.6

**^a^** Crystal size calculated from the XRD diffraction peaks of CeO_2_ (111) based on the Scherrer equation.

**Table 3:** Surface concentration of Ce cations and oxygen vacancies determined with XPS.

Catalysts	Ce Atom Ratio (%)		O Atom Ratio (%)
	Ce^3+^	Ce^4+^	O_V_
CeO_2_	13.5	86.5	5.3
CeO_2_–H450	15.7	84.3	6.9
CeO_2_–H550	16.2	83.8	7.6
CeO_2_–H650	16.7	83.3	7.8
CeO_2_–H750	16.8	83.2	8.1
CeO_2_–H800	17.7	82.3	8.5
CeO_2_–H550–O350	15.7	84.3	7.1
CeO_2_–H550–O450	15.3	84.7	6.6
CeO_2_–H550–O550	15.2	84.8	6.7
CeO_2_–H750–O350	15.8	84.2	7.2
CeO_2_–H750–O450	15.2	84.8	6.5
CeO_2_–H750–O550	14.9	85.1	6.1

**Table 4:** Adsorption amount of CO_2_ and NH_3_ determined using TPD profiles.

Catalysts	CO_2_ Adsorption (mmol/g)			NH_3_ Adsorption (mmol/g)		
	Weak (<200 °C)	Moderate (200~400 °C)	Strong (>400 °C)	Weak (<200 °C)	Moderate (200~400 °C)	Strong (>400 °C)
CeO_2_	0.607	0.206	0.039	0.592	0.158	0.040
CeO_2_–H450	0.533	0.147	0.025	0.472	0.124	0.046
CeO_2_–H550	0.525	0.136	0.020	0.586	0.134	0.043
CeO_2_–H650	0.361	0.074	0.015	0.384	0.084	-
CeO_2_–H750	0.142	0.023	0.002	0.199	0.018	-
CeO_2_–H800	0.113	0.019	0.006	0.117	0.031	-
CeO_2_–H550–O350	0.525	0.130	0.024	0.586	0.143	0.040
CeO_2_–H550–O450	0.538	0.154	0.028	0.416	0.143	0.037
CeO_2_–H550–O550	0.590	0.156	0.028	0.598	0.152	0.041
CeO_2_–H750–O350	0.183	0.023	0.004	0.227	0.022	-
CeO_2_–H750–O450	0.192	0.024	0.006	0.232	0.023	-
CeO_2_–H750–O550	0.205	0.039	0.007	0.240	0.027	-

## Data Availability

All the relevant data used in this study have been provided in the form of figures and tables in the published article, and all data provided in the manuscript are available to whom they may concern.

## Supplementary Materials

The following supporting information can be downloaded at https://www.mdpi.com/article/10.3390/molecules28093785/s1, Figure S1: Change in the color of catalysts.
