# Supplementary material for: Elucidating the Role of Surface Ce4+ and Oxygen Vacancies of CeO2 in the Direct Synthesis of Dimethyl Carbonate from CO2 and Methanol
Source: Molecules. 2023 Apr 28;28(9):3785. doi: 10.3390/molecules28093785 (PMC10180377; doi:10.3390/molecules28093785)
Supplement: Supplementary file 1 [file molecules-28-03785-s001.zip › molecules-2300523-supplementary.pdf]

Elucidating the role of surface Ce^{4+} and oxygen vacancy of CeO_2 for direct synthesis of dimethyl carbonate from CO_2 and methanol

Guoqiang Zhang[1*], Yuan Zhou[1, 2], Yanlin Yang[1], Tiantian Kong[1], Ya Song[1], Song Zhang[1] and Huayan Zheng[1*]

[1] Department of Food Science and Engineering, Moutai Institute, Renhuai 564502, China
[2] Experimental Training Teaching Center, Moutai Institute, Renhuai 564502, China
* Correspondence: zgq198615@163.com (G. Zhang), andyzheng1109@163.com (H. Zheng)

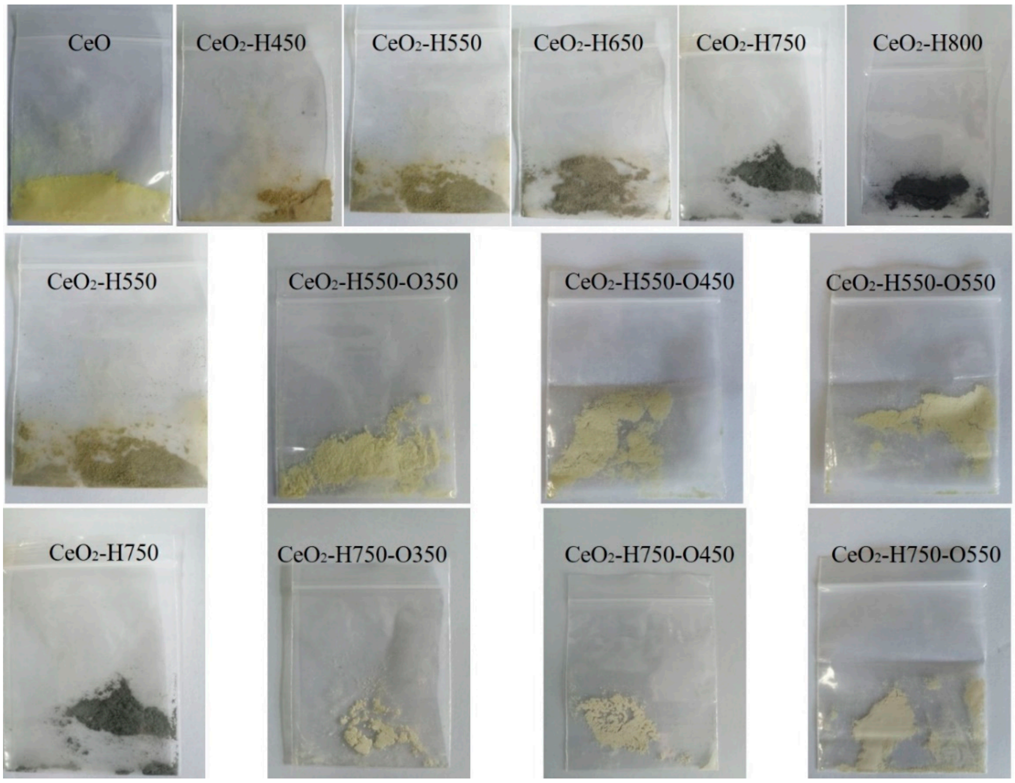

Figure S1. The change of color of catalysts
